# Common and differential features of liver and pancreatic cancers: molecular mechanism approach 

**Published:** 2021

**Authors:** Kourosh Saki, Vahid Mansouri, Nastaran Asri, Mohammad Fathi, Zahra Razzaghi

**Affiliations:** 1 *Proteomics Research Center, Faculty of Paramedical Sciences, Shahid Beheshti University of Medical Sciences, Tehran, Iran*; 2 *Gastroenterology and Liver Diseases Research Center, Research Institute for Gastroenterology and Liver Diseases, Shahid Beheshti University of Medical Sciences, Tehran, Iran*; 3 *Critical Care Quality Improvement Research Center, Faculty of Paramedical Sciences, Shahid Beheshti University of Medical Sciences, Tehran, Iran*; 4 *Laser Application in Medical Sciences Research Center, Shahid Beheshti University of Medical Sciences, Tehran, Iran*

**Keywords:** Pancreas, Ductal adenocarcinoma, Liver cancer, Network analysis, Biomarker

## Abstract

**Aim:**

The aim of this study was to introduce biomarkers commonly involved in pancreatic cancer metastasis to the liver.

**Background:**

The liver is affected by metastatic disease in pancreatic cancer.

**Methods:**

Two cancer biomarkers were distinguished through a STRING database protein query. The dysregulated proteins of the two cancers were included in 2 networks drawn by Cytoscape software v 3.2.7. 20 top nodes and achieved by the Network analyzer application of Cytoscape based on degree value. The common hub nodes were determined, and action maps were analyzed.

**Results:**

Among 20 hubs of each studied cancer, 18 common hub nodes (90% of hubs) were identified and screened by action maps. Four proteins, AKT1, CDKN2A, ERBB2, and IL6, were identified as common central proteins related to the two studied diseases.

**Conclusion:**

AKT1, CDKN2A, ERBB2, and IL6 are common protein core of liver and pancreatic cancers, while STAT3, CASP3, NOTCH1, and CTNNB1 are possible differential proteins to discriminate these cancers.

## Introduction

 Pancreatic adenocarcinoma (PAC) is one of the most aggressive human malignancies and a major cause of cancer mortalities in the world (1). About 50% of patients with pancreatic cancer have liver metastasis with a poor prognosis (2). It has been widely confirmed that metastasis determines the prognosis of PAC, and the primary distant location of PAC is the liver (3). Median survival time for pancreatic cancer patients with metastatic liver treated with current methods is less than 6 months (4, 5). Liver metastasis cannot be resected in most cases; however, survival prolongation methods such as chemotherapy and radiation therapy in addition to controlling quality of life is the aim of treatment in such cases (6, 7). Assessment of highly accurate biomarkers for early detection of PAC could impact patients’ prognoses to 5 years survival after incidental diagnosis of small tumors confined to the pancreas (8). Exploring genetic liver-related metastatic markers to predict clinical outcome in PAC patients is valuable (3). Complicated steps are involved in PAC metastasis to the liver, such as adhesion of dissociated PAC cells toward the liver, remodeling of extracellular matrix, immune escape, and micro-metastasis angiogenesis (9). Pancreatic tumors are highly heterogenous in cellular and molecular levels (10), and molecular biomarkers are useful tools for determining PAC prognoses (11). Multiple prognostic models with predictive value for PCA have been established based on public data banks such as Cancer Genome Atlas (CGA) and Gene Expression Omnibus (GEO) (12). Proteomics mass spectrophotometry-based (MS) analysis is a powerful technology for extracting remarkable amounts of clinical information from blood sera of PAC patients in an untargeted manner in addition to information accessible from the databanks. PPI network analysis to find new biomarkers for assessment of different diseases is an attractive subject for scientists (13). High throughput clinical proteomics methods and network analysis of the function of genes and proteins related to different aspects of PAC are also of great interest to researchers (14). The upregulated genes ANO1, FAM83A, GPR87, ITGB6, KLK10, and SERPINE1 with the downregulated SIMIM32 protective gene were introduced as prognostics for liver metastatic conditions (3). KRAS mutation status is considered as a biomarker and may act as a prognostic factor for unresectable pancreatic cancer (15). TP53, KRAS, SMAD4 (DPC4), and P16 (CDKN2) genes mutation in pancreatic adenocarcinoma has been reported by Cowgill et al. (16). Tavirani et al. introduced TP53, AKT1, EGFR, EGF, MYC, and HRAS as key node genes in various cancers such as pancreas adenocarcinoma (17). Network analysis of PPI in hepatocellular carcinoma (HCC) revealed that the metabolism and cytoskeletal biological process involved in HCC and cancer related proteins, such as SRC and PKM2, are hub nods. Qin et al. used survival rate analysis to introduce PKM2 and ARPC4 proteins as potential prognostic markers of HCC (18). KLFL2 and CXCL12 are suggested for progression of pancreatic neuroendocrine tumors (19). Seo et al. revealed that VEGF is an important predictor for liver metastasis and poor prognosis in ductal pancreatic adenocarcinoma (20). Poorly differentiated PAC as a lethal neoplasm requires early detection, and Lu et al. have suggested *CEACAM5, KRT6A, KRT6B, KRT7, *and* KRT17 genes as prognostic biomarkers (*21*). *Total IGF-2 and IGFBP-2 are suggested as predictive markers for identifying metastatic PAC patients treated with gemcitabine (22). Considering the variety of biomarkers that have been cited and sometimes introduced in various studies, it is clear that biomarkers that clearly indicate pancreatic cancer and liver metastasis have not yet been identified. The intent of this study was to introduce common biomarkers related to pancreatic cancer that metastasizes to the liver with the aid of databases and data analysis software. 

## Methods

Two scale free networks were formed for liver and pancreatic cancers. Among 20 hubs of each network, 18 of them were common, comprising GAPDH, TP53, EGFR, MYC, INS, ALB, IL6, AKT1, VEGFA, CDH1, PTEN, EGF, CDKN2A, KRAS, ESR1, CCND1, ERBB2, and HRAS (Table 1). 

**Table 1:** List of 18 common hub nodes between liver and pancreatic cancers. K liver and K pancreas refer to degree values in the networks of liver and pancreas respectively. Proteins are listed as degree value of nodes in liver cancer

No.	Name	K liver	K Pancreas
1	GAPDH	82	77
2	TP53	82	84
3	EGFR	80	81
4	MYC	80	78
5	INS	79	76
6	ALB	79	74
7	IL6	78	75
8	AKT1	78	78
9	VEGFA	78	78
10	CDH1	76	77
11	PTEN	75	76
12	EGF	75	79
13	CDKN2A	75	81
14	KRAS	73	82
15	ESR1	73	76
16	CCND1	72	72
17	ERBB2	72	79
18	HRAS	76	83

**Figure 1:**
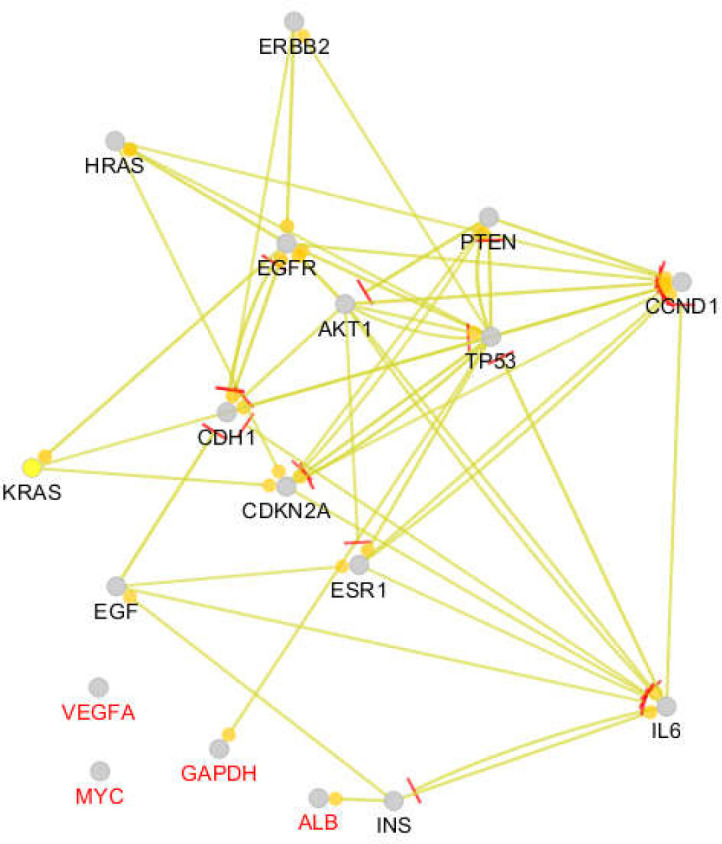
Expression map of the 18 introduced common hub nodes of liver and pancreatic cancers networks. The yellow and round tips refer to upregulation action and the red tips correspond to downregulation. The nodes which are labeled in red color only were regulated or isolated

**Figure 2:**
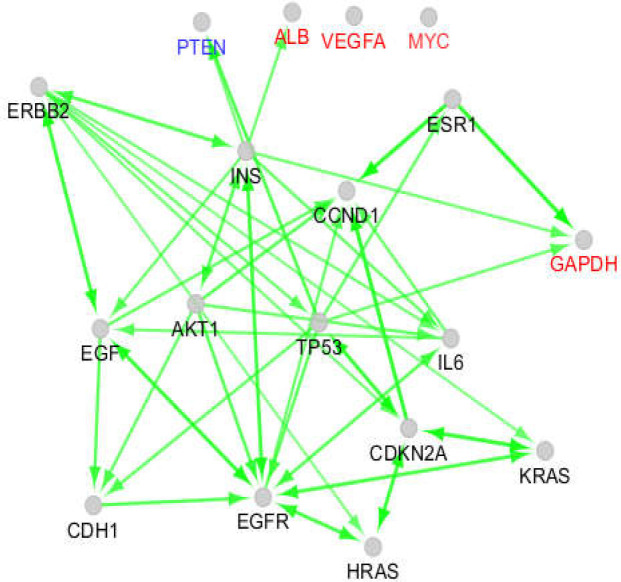
Activation map of the 18 introduced common hub nodes of liver and pancreatic cancers networks. Direction of arrow refer to activation. The nodes which are labeled in red color are the individuals that only were regulated or isolated in expression map. PTEN is a node which only is activated

**Figure 3:**
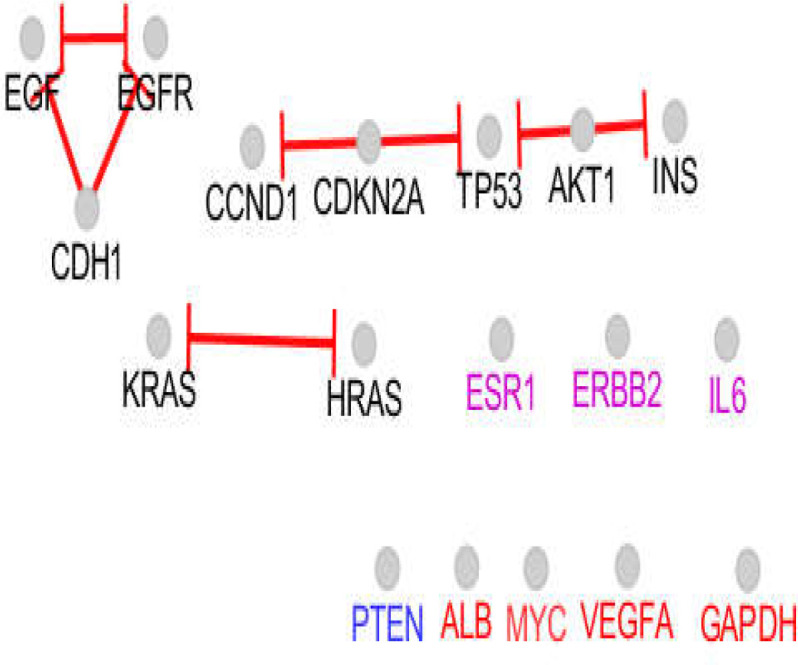
inhibition map of the 18 introduced common hub nodes of liver and pancreatic cancers networks. Bar tips refer to inhibition. The nodes which are labeled in red color are the individuals that only were regulated or isolated in expression map. PTEN is a node which only is activated in the action map. ESR1, ERBB2, and IL6 as like PTEN, ALB, MYC, VEGFA, and GAPDH are isolated

STAT3 and CASP3 were determined as specific hub nodes related to liver cancer, while NOTCH1 and CTNNB1 were characterized as the specific hubs of pancreatic cancer. An expression map of the 18 introduced common hub nodes is presented in Figure 1. The hubs that had no regulatory effect on the other hubs were differentiated from the other individuals. The activation and inhibition actions of the common hubs are presented in Figures 2 and 3. As shown in Figure 2, the hubs that had no regulatory effect on the expression map plus PTEN only are regulated by the other individuals and are not regulators of other proteins. Based on the inhibition map (see Figure 3), the inhibitor and inhibited hubs are restricted to 10 of the hub nodes. 

## Results

Two scale free networks were formed for liver and pancreatic cancers. Among 20 hubs of each network, 18 of them were common, comprising GAPDH, TP53, EGFR, MYC, INS, ALB, IL6, AKT1, VEGFA, CDH1, PTEN, EGF, CDKN2A, KRAS, ESR1, CCND1, ERBB2, and HRAS (Table 1). STAT3 and CASP3 were determined as specific hub nodes related to liver cancer, while NOTCH1 and CTNNB1 were characterized as the specific hubs of pancreatic cancer. An expression map of the 18 introduced common hub nodes is presented in Figure 1. The hubs that had no regulatory effect on the other hubs were differentiated from the other individuals. The activation and inhibition actions of the common hubs are presented in Figures 2 and 3. As shown in Figure 2, the hubs that had no regulatory effect on the expression map plus PTEN only are regulated by the other individuals and are not regulators of other proteins. Based on the inhibition map (see Figure 3), the inhibitor and inhibited hubs are restricted to 10 of the hub nodes.

## Discussion

There are many documents that discuss the relationship between liver and pancreatic cancers (25-27). In this study, it was expected that a high similarity between the molecular mechanisms of these two cancers would be found. The results showed that 90% of hub nodes were common between the networks of these two diseases. Attempts were made to screen the 18 common hubs to identify a limited numbers of critical proteins which promote the mechanisms of the two studied diseases. The expression map revealed that VEGFA and MYC were excluded from the nodes which formed the network. These two proteins were isolated in the activation and inhibition maps. It can be concluded that VEGFA and MYC cannot play a critical role in the development of the two studied cancers. ALB and GAPDH, the other common hub nodes, had no regulatory effect on the expression map and are upregulated by INS and TP53, respectively. ALB is activated by INS and is isolated in activation and inhibition maps; however, GAPDH is activated by TP53, ESR1, and INS and remained isolated in the inhibition map, and it had no activation effect in the action map. It seems that GAPDH and ALB are not suitable candidates for further investigation. 

As depicted in all action maps, TP53 plays a central role and is connected to the important elements of the networks. Based on the results shown in Table 1, TP53 is a top hub node for liver and pancreatic cancers. Since TP53 as a common tumor marker is related to many cancers (28), it is not a suitable protein for further assessment in this study, but TP53 regulators can be considered as critical hubs. As shown in Figure 3, TP53 is inhibited by AKT1 and CDKN2A. ERBB2 and CDKN2A activate TP53 (see activation map). TP53 is upregulated by AKT1 and IL6, however, AKT1 also downregulates TP53.

The current findings indicate that AKT1, CDKN2A, ERBB2, and IL6 are the critical common hubs related to promoting both liver and pancreatic cancers. AKT1 is one of the serin threonine kinases which regulates many cell functions such as proliferation, survival, and cell metabolism through serin threonine phosphorylation. AKT1 is one of the central proteins in the PI3K/Akt signaling pathway that mediates several cellular processes such as proliferation, motility, growth, and survival (29). Increased activity of AKT in many cancer types has been demonstrated with antiapoptotic survival signal transmission (30). Increased deregulation and mutation of PI3K/Akt pathway was implicated in tumorigenesis and confers resistance to chemotherapy (31, 32). Akt activation in pancreatic cancer is a frequent event correlated with the presence of phosphorylated Akt, which is associated with worse prognostic variables (33, 34). Inhibition of the PI3K/Akt pathway sensitizes pancreatic cells to the apoptotic effects of chemotherapy (35). Ductal pancreatic adenocarcinoma KRAS mutant revealed early metastasis to the liver. Akt1 activity in combination with KRAS oncogenic mutant could accelerate pancreatic progression toward invasive pancreatic ductal adenocarcinoma (36). The CDKN2A gene encodes the p16 protein that inhibits cyclins to inhibit immature transition from G1 to S phase, serving as a tumor suppressor (37). Increased risk of pancreatic cancer is observed in cases with CDKN2A mutations in familial melanoma (38). However, research has revealed that germ line mutation of CDKN2A could lead to familial pancreatic cancer with low prevalence in populations (39). Loss of CDKN2A could lead to uncontrolled cell proliferation (40). It is commonly believed that activation of the p16 protein in patients with primary liver cancer is related to homozygous deletion and mutation of the CDKN2A gene (41, 42). Methylation of the CDKN2A gene has been extensively reported in hepatic cancer patients (43) accompanied by gene methylation in chronic liver disease patients (44). ERBB2, or HER2, is a receptor tyrosine kinase, and its overexpression is associated with poor prognosis in pancreatic cancer (45). ErBb2 is strongly upregulated in hepatitis B-infected livers and has been suggested as an early marker of hepatocellular carcinoma (46). IL6 is a potent proinflammatory cytokine which is elevated in patients with pancreatic cancer and potentially increases tumor cell invasion in vitro (47). Pancreatic cancer promotes the formation of a pro-metastatic niche in the liver by secreting IL6 produced by non-cancer cells within pancreatic tumors, which activate proinflammatory STAT3 signaling in hepatocytes. Lee and Beaty believed that general inflammation induced by IL6 was sufficient to establish an environment conducive to the spread of cancer cells in the liver, even in the absence of cancer (48). The current results confirm the findings of studies identifying AKT1, CDKN2A, ERBB2, and IL6 as critical markers for metastasis of pancreatic and liver cancers. 

The current findings indicate that STAT3 and CASP3 are related to liver cancer, while NOTCH1 and CTNNB1 are associated with pancreatic cancer. The signal transducer and activator of transcription 3 (STAT3) as a transcription factor and constitutive activation of SATA3 by phosphorylation of Tyr70S were reported in several human tumors, such as pancreatic ductal adenocarcinoma (49, 50). This finding is in conflict with the current results. It should be mentioned that the central nodes are discussed here, and STAT3 as a non-central protein may be related to PAC. STAT3 controls acute phase genes in response to IL6 and EGF during inflammation (51). IL6 is a stimulator of STAT3 activation, and a decline in circulating IL6 may serve as an indicator of STAT3 pathway inhibition (52). Nuclear factor-kappa B in the NF-kB/IL-6/STAT3 pathway plays an important oncogenic role in cancers that arise from chronic inflammation such as hepatocellular carcinoma (53). CASP3 is a downstream effector of cytosine protease in apoptosis, and it is frequently overexpressed in hepatocellular carcinoma associated with high serum levels of AFP (54). CASP3 expression does not correlate with cell apoptosis in hepatocellular carcinoma (55). Notch1 is reported to be a key member involved in pancreatic carcinogenesis-mediated TGF-α-induced changes during pancreatic tumorigenesis (56). Notch1 expression could be detectable in pancreatic ductal adenocarcinoma cases, and Notch1/Hes1 signaling is reactivated; however, Notch1 as a feto-oncoprotein is activated during organogenesis and carcinogenesis (57). CTNNB1 encoding of B-catenin and Wnt/B-catenin aberrant signaling could lead to the development of cancers such as controversial pancreatic ductal adenocarcinoma (58). Immunohistochemistry and polymerase chain reaction confirmed Wnt/B-catenin signaling pathway upregulation in pancreatic ductal adenocarcinoma (59, 60). 

The current results indicate that AKT1, CDKN2A, ERBB2, and IL6 are the common protein core of liver and pancreatic cancers, and STAT3, CASP3, NOTCH1, and CTNNB1 are possible differential proteins that discriminate these cancers. STAT3 and CASP3 were dysregulated in liver cancer; however, NOTCH1 and CTNNB1 were related to pancreatic cancer. These findings represent a new perspective on the two discussed diseases. Further investigations based on patient information is suggested to provide supportive data.

## Conflict of interests

The authors declare that they have no conflict of interest.
